# Age and Environment Influences on Mouse Prion Disease Progression: Behavioral Changes and Morphometry and Stereology of Hippocampal Astrocytes

**DOI:** 10.1155/2017/4504925

**Published:** 2017-01-24

**Authors:** J. Bento-Torres, L. L. Sobral, R. R. Reis, R. B. de Oliveira, D. C. Anthony, P. F. C. Vasconcelos, Cristovam Wanderley Picanço Diniz

**Affiliations:** ^1^Laboratório de Investigações em Neurodegeneração e Infecção, Hospital Universitário, João de Barros Barreto, Instituto de Ciências Biológicas, Universidade Federal do Pará, Belém, PA, Brazil; ^2^Universidade do Estado do Pará, Centro de Ciências da Saúde, Belém, PA, Brazil; ^3^Vertebrate Embryology Laboratory, Biomedical Sciences Institute, Health Sciences Center, Federal University of Rio de Janeiro, Rio de Janeiro, RJ, Brazil; ^4^Lab of Experimental Neuropathology, Department of Pharmacology, University of Oxford, Oxford, UK; ^5^Departamento de Arbovirologia e Febres Hemorrágicas, Instituto Evandro Chagas, Ananindeua, PA, Brazil

## Abstract

Because enriched environment (EE) and exercise increase and aging decreases immune response, we hypothesized that environmental enrichment and aging will, respectively, delay and increase prion disease progression. Mice dorsal striatum received bilateral stereotaxic intracerebral injections of normal or ME7 prion infected mouse brain homogenates. After behavior analysis, animals were euthanized and their brains processed for astrocyte GFAP immunolabeling. Our analysis related to the environmental influence are limited to young adult mice, whereas age influence refers to aged mice raised on standard cages. Burrowing activity began to reduce in ME7-SE two weeks before ME7-EE, while no changes were apparent in ME7 aged mice (ME7-A). Object placement recognition was impaired in ME7-SE, NBH-A, and ME7-A but normal in all other groups. Object identity recognition was impaired in ME7-A. Cluster analysis revealed two morphological families of astrocytes in NBH-SE animals, three in NBH-A and ME7-A, and four in NBH-EE, ME7-SE, and ME7-EE. As compared with control groups, astrocytes from DG and CA3 prion-diseased animals show significant numerical and morphological differences and environmental enrichment did not reverse these changes but induced different morphological changes in GFAP+ hippocampal astroglia. We suggest that environmental enrichment and aging delayed hippocampal-dependent behavioral and neuropathological signs of disease progression.

## 1. Introduction

Prion diseases are fatal neurodegenerative diseases characterized by accumulation of prion misfolded (PrP^sc^) protein, gliosis, synaptic dysfunction, and, at late stages, neuronal loss [1–5]. This sequence of events shares the neuropathological features of chronic neurodegeneration in Alzheimer's disease (AD) and is well reproduced in the ME7 murine young adult model of prion disease [6–8]. Previous studies in murine model of prion disease identified as disease progresses an increase in immunoreactivity of GFAP (glial fibrillary acidic protein), a selective marker for astrocytes [9]. The contribution of astrocytes in young adult scrapie pathogenesis at the cellular and molecular levels has been recently investigated and resulted in a better understanding of disease progression mechanisms [10]. Prion disease is caused by a conformational change of the normal membrane bound glycoprotein PrPc (cellular prion protein) into a insoluble infectious form PrPSc (scrapie isoform) [11]. A close relationship between PrPSc and enhanced glial fibrillary acidic protein (GFAP) immunoreactivity at different stages of the disease has been identified [3, 12], giving morphological evidence of specific astrocytic involvement in the progression of prion disease. In addition, a recent analysis of the hippocampal proteome in ME7 prion disease in correlation with behavioral and cellular dysfunctions at different time points has revealed a predominant astrocytic signature with four upregulated proteins, including GFAP, selectively expressed in astrocytes [4].

Prion pathogenesis is, however, dramatically slowed in aged mice when compared with young animals [13, 14]. Indeed, aged infected mice show significantly less disease-specific pathological markers such as gliosis, vacuolation, and PrP^sc^ and less pronounced upregulation of disease-associated inflammatory and stress-response genes [15]. These findings suggest that an immune senescent system may contribute to slowing down neuropathological and behavioral changes. A healthy, young, fully responsive immune system, therefore, may be necessary to generate the whole spectrum of prion disease features in rapid progression.

Previous reports demonstrated beneficial effects of environmental enrichment on neurodegenerative disease progression in experimental models including Huntington's disease [16–18], transgenic mice coexpressing familial AD-linked APP and PS1 variants [19], MPTP and 6-OHDA Parkinson's disease models [20, 21], and amyotrophic lateral sclerosis mouse model expressing the human SOD1(G93A) gene mutation [22]. However, no previous study has investigated the possible beneficial effects of EE on prion disease outcomes.

Both environmental changes and aging influence astrocytic plasticity [23–26], which seems to be a key element of the host protective system in prion progression [3, 4, 27]. For this reason, we evaluated possible correlations between behavioral changes related to prion disease progression and number and morphology of astrocytes in both young adult and aged prion-diseased mice. Because we previously demonstrated in albino Swiss mice model of prion disease that astrocytes from the polymorphic layer of dentate gyrus exhibit intense reactive astrocytosis earlier than all other layers of dentate gyrus and that this change was associated with axonal degeneration of mossy fiber, we thought polymorphic layer and CA3 (target of mossy fibers) may exhibit the earliest astroglial pathology of the hippocampal formation of albino Swiss mouse model of prion disease. In addition, to the selected areas for morphometry and stereological analysis of astrocytes, we have chosen a time window for astrocytes reconstructions when stereological data showed no change in the number of neuronal cell bodies [27].

Stereological analysis and microscopic three-dimensional reconstruction combine unbiased systematic sampling approach to count cells [28] and detailed morphological description of objects of interest [29]. This combination increases resolution of quantitative neuropathology and offers an integrative analysis of morphology and numeric cell changes using hardware and software readily available. Indeed, these new analytic tools in correlation with clinical outcomes in different time windows may provide reliable detailed quantitative information about subtle or profound changes in abnormal conditions that may help to understand pathogenesis [30, 31]. Here we used this unbiased meticulous approach to investigate influences of age and environment on morphology and numbers of astrocytes of hippocampus in a murine model of prion disease.

## 2. Methods

All procedures were approved by the institutional Animal Care Committee of the University Hospital of the Federal University of Pará under the Protocol Number 1701/05. All animals were handled in accordance with the “Guidelines for the Use of Animals in Research” and followed the legal requirements of the Brazilian Council of Experimental Animal Research (CONCEA). A total of 33 albino Swiss female mice at ages 6 (young) and 18 (aged; A) months were used. Adult males could not be used because of aggression levels [32]. Females were housed in EE or standard environment (SE) cages for 5 weeks, followed by surgeries for intracerebral injection of ME7-infected or normal brain homogenates (NBH). After recovery from anesthesia, all animals were returned to their preoperative housing and tested weekly for burrowing activity. At 18th week after injection (wai), all mice were subjected to the object recognition test and then euthanized and perfused.

### 2.1. Housing, Food, and Welfare

EE animals (*n* = 12; 6 ME7, 6 NBH) were housed in plastic cages (32 × 39 × 16.5 cm) equipped with wheels for voluntary running, tunnels, and various plastic, metal, or cardboard objects that were exchanged and/or placed at different positions once a week to stimulate exploratory activity. SE mice (*n* = 21; 5 NBH, 6 ME7; 4 NBH-A, 6 ME7-A) were kept in a similar type of cage but with minimal environmental stimulation. Both cage types were lined with autoclaved rice straw, which was exchanged at least weekly. Animals had free access to food (containing 23 g protein/100 g) and water and were maintained on a room with minimum noise exposure, at 23 ± 2°C, 12 h light/dark cycle and light period was used for behavioral tests. Because we have not investigated possible environmental influence on aged mice prion disease progression, no data from aged mice raised on enriched cages are exhibited.

### 2.2. Intracerebral Inoculation

**Figure 1:**
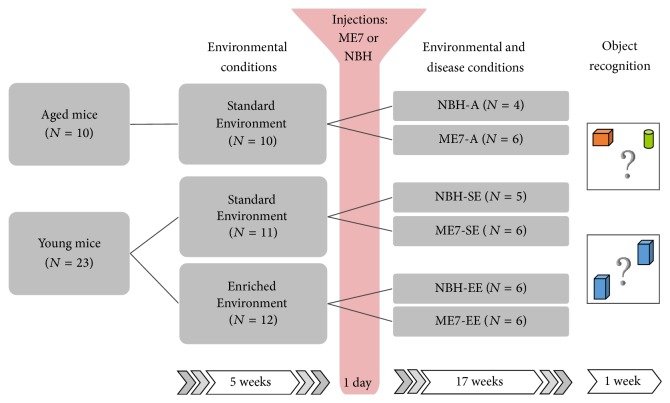
Experimental timeline. Young adult (6 months old) and aged (18 months old) female albino Swiss mice were maintained for 5 weeks either in enriched or in standard cages and then subjected to injections of normal or ME7 infected brain homogenates and returned to their original cages. After 17 weeks after injections they were submitted to placement and identity object recognition tests, euthanized, and had their brains processed for selective GFAP, PrPSc, and IBA1 immunolabeling.

After the 5-week cage acclimation, animals were weighed and i.p. anesthetized with 2,2,2-tribromoethanol (Sigma-Aldrich, USA) (250 mg/kg body weight) for stereotaxic surgery (Insight Equipment) for bilateral intracerebral inoculation of 1 *μ*L of normal (NBH) or ME7-infected brain homogenates. ME7 tissue was obtained from mice with clinical signs of prion terminal illness and kindly provided by Professor V. H. Perry (Centre for Biological Sciences, University of Southampton, Southampton, UK). Because integrity of hippocampal formation would be essential to guarantee fine details of astrocytes three-dimensional reconstruction and mechanical damage induced by injections needle could induce reactive astrogliosis around the tract, we decided to select dorsal striatum as the target area of injections. Thus, injections were made on the following coordinates: +1 mm bregma, ±1.5 mm from midline, and 3.0 mm deep [33]. Two openings were made with the aid of a drill to enable bilateral striatal injections (10% w/v in phosphate buffered saline (PBS)) of NBH or ME7-infected homogenates. After injection, the needle (Hamilton 10 *μ*L) was held in place for 3 minutes to avoid solution backflow, after which it was slowly withdrawn. The scalp was then sutured and the animal put into a cage for recovery. After recovery, animals were returned to their original cages. Ultimately, the numbers for treatment and control groups were as follows: NBH-SE = 5; NBH-EE = 6; ME7-SE = 6; ME7-EE = 6; NBH-A = 4; and ME7-A = 6. As noted, at 18 wai, all animals were perfused for brain tissue analysis.  Figure 1 is a graphic representation of the experimental timeline.

### 2.3. Behavior Assessment

#### 2.3.1. Burrowing

Burrowing is the most sensitive test to indicate earlier signs of hippocampal prion-induced dysfunction [34]. In this test a tube with food pellets is placed in the cage of a single mouse and almost all mice spontaneously remove pellets out of the tube. It is expected that a normal mouse will burrow many times its own weight in food pellets in two hours [35]. Weekly for a 4-hour testing period (from 09:00 to 11:00 hours), each animal was individually placed in a plastic cage (32 cm × 39 cm × 16.5 cm) containing a cylindrical PVC tube (20 cm length, 7.2 cm diameter) filled with 250 g of normal diet food pellets. The open end of the tube was supported 3 cm above the floor. After the testing period, the remaining food in the cylinders was weighed, and the animals were returned to their collective cages [34]. Burrowing measurements started at week 3 after injection and finished at 18 weeks.

#### 2.3.2. Placement and Object Identity Recognition

We used one-trial object placement recognition [36] and one-trial identity recognition tests [37]. All mice were subjected to both tests.

The apparatus for these tests consisted of an open box (30 × 30 × 40 cm) made of painted white wood. The floor was painted with black lines to form nine squares (10 × 10 cm), and the luminance at the center of the cage floor was 2.4 cd/m^2^. One meter above the open field, a video camera connected to a computer recorded each training session for later analysis by Any-Maze software (Stöelting). Detailed protocols and the rationale for test choices are discussed elsewhere [38]. In brief, behavioral tests were performed after 12 days, with 7 days to become accustomed to handling, 3 days for open field habituation, and 2 days for object habituation, with testing on the 13th day.

For handling, mice were daily picked up by the tail and released into the center of the open field. After 1 min of open field exposure, the animals were removed from the open field and placed back into their home cages. For open field habituation, mice were placed daily in the arena, free of objects, for 5 minutes to explore the open field. To achieve object habituation, mice were exposed to two identical objects placed at the corners of the arena for 5 minutes, three times, with 50 minutes in between. These objects were not used on the test days. Finally, on test day, memory tests were administered once for each mouse.

To minimize the influence of natural preferences for particular objects or materials, we chose objects of the same material, with different geometries that could be easily discriminated, and similar access for interaction [39]. All objects were made of plastic with different shapes, heights, and colors. Before each mouse entered in the arena, the box and objects were cleaned with 75% ethanol to minimize distinguishing olfactory cues.

On the testing day, in a first trial, mice were exposed for 5 minutes to two identical objects (samples) and, in a second trial of 5 minutes, to two dissimilar objects, one a familiar object (from the sample session) and the other a new distinct object. The intertrial interval was 50 minutes. Most mice were expected to spend more time with the novel than the familiar object. Similarly, the next day, animals were subjected to the one-trial placement recognition test. In this test, mice were exposed for 5 minutes in the first trial to two identical objects. After an intertrial interval of 50 minutes, mice were exposed for 5 minutes to the same objects, one stationary and one displaced. The expectation was that, after the intertrial interval, most mice would spend more time with the displaced than the stationary object.

We measured the time of exploration on each object, expressed as a proportion (percentage) of the total time of exploration. Possible significant differences were also detected with the two-tailed *t*-tests for dependent groups [40].

### 2.4. Perfusion and Histology

At week 18 after injection, when ME7-Y mice show typical behavioral signs of preclinical disease, including a significant reduction in burrowing activity and increase in locomotor exploratory activity [35], they were weighed, i.p. anesthetized with 2,2,2-tribromoethanol (Sigma-Aldrich, USA) (250 mg/kg body weight), and perfused transcardially with heparinized 0.9% saline solution (5000 IU/L), followed by 4% paraformaldehyde in 0.1 M phosphate buffer (pH 7.2–7.4). After perfusion, the brains were removed and sectioned in a vibratome (Micron) on the horizontal plane (70 *μ*m thick); see detailed histological procedures elsewhere [27]. Serial anatomical series of sections (1 : 6) were collected and subjected to immunohistochemical analysis, as described below.

#### 2.4.1. Immunohistochemistry for GFAP and Iba1

The morphology and number of astrocytes were assessed using the primary antibody to GFAP (Millipore© # MAB360) and Iba1 (Wako© # 019-19741). Brain sections from both diseased and control animals were selected randomly and systematically, taking one in every six slices of 70 *μ*m. The sections were washed in 0.1 M phosphate buffer, pH 7.2–7.4, and pretreated for 60 minutes in 0.2 M boric acid solution, pH 9.0, at 70°C for antigenic recovery. After being washed with PBS-5% Triton, sections were transferred for 15 minutes to a solution of 0.3% hydrogen peroxide in 0.1 M phosphate buffer, pH 7.2–7.4. After washing sections in PBS, we followed the protocol for the Mouse-on-Mouse Kit (Vector Laboratories), using anti-GFAP or anti-Iba1 primary antibodies at 1 : 400 in 0.1 M PBS, pH 7.2–7.4.

Finally, sections were washed in PBS and incubated in ABC (Avidin-Biotin Complex Vector Laboratories) for 30 minutes. The DAB/nickel/glucose oxidase/diaminobenzidine protocol was used to reveal GFAP and Iba1 binding sites [41]. Sections were mounted on gelatinized slides, left to dry at room temperature, and subsequently counterstained with cresyl violet, dehydrated in alcohol (70, 80, 90, and 100%), and cleared in xylenes before being covered with Entellan (Merck) and cover slips for further analysis.

#### 2.4.2. Protease-Resistant PrP^c^ Immunolabeling

Sections immunoreacted to detect PrP protease resistance were pretreated in formic acid 85%, for 30 minutes, incubated in trypsin 0.05% at room temperature for 10 minutes, and then transferred to a solution of 0.1 M citrate buffer, pH 6.0, at 90°C for 1 hour. Sections were rinsed in 0.1 M PBS-Triton X-100 (5%). After a wash in PBS, the sections were subjected to the Mouse-on-Mouse (MOM) Blocking Kit (Vector Laboratories, Burlingame, CA, USA) protocol, as follows: MOM IgG blocking for one hour, primary antibody exposure for 72 hours (ABCAM, mouse monoclonal [8H4] to Prion protein PrP, 1 : 2500) diluted in 0.1 M PBS, and washes in 0.1 M PBS three times for 5 minutes, followed by MOM Biotinylated Anti-Mouse IgG Reagent for 12 hours. Sections were treated with 0.3% hydrogen peroxide in 0.1 M phosphate buffer, pH 7.2–7.4, then transferred to ABC solution for 1 hour, and washed again before incubation in acetate buffer 0.2 M, pH 6.0, for 5 minutes and revealed in GND solution (diaminobenzidine 0.5 mg/mL, ammonium nickel chloride 0.6 mg/mL, and glucose oxidase). All steps were carried out under gentle and constant agitation.

### 2.5. Stereology

The quantitative analysis of the number of objects of interest in regions of interest in infected mice or controls from the different environmental conditions was performed using the optical fractionator method [42]. The regions of interest were the CA3 stratum radiatum and the polymorphic layer of the dentate gyrus (DG) of the dorsal hippocampal formation. Their boundaries were defined using a 4x objective optical microscope (Nikon Eclipse 80i, Japan) equipped with a motorized stage to control the *X*, *Y*, and *Z* coordinates with help of a stage controller (MAC6000, Ludl Electronic Products, Hawthorne, NY, USA). This system was coupled to a computer containing the Stereo Investigator program (MBF Bioscience, Williston, VT, USA), which recorded the three-dimensional coordinates and stored the stereological data. A 100x objective was used to count and reconstruct astrocytes. The stereological parameters for counting astrocytes were dissector 12, guard 1, and counting probe 80–60 for the CA3 and DG regions, with a sample grid 130–100 for CA3 and 100–80 for DG.

Astrocyte cell body volume was obtained during the counting procedures using an optical fractionator. The satellite probe “nucleator,” when run in parallel with the optical fractionator, allows each cell to have the same probability of being selected regardless of shape, orientation, or size [43]. The analysis consisted of the superposition of six radial lines (the number of lines configured according to interest) that crossed the cell body in the center of the point marked by counting the optical fractionator. The user identified the point at which each radial line intersected the edge of the cell body, and the spreadsheet program provided the results for volume, area, and the coefficient of error.

### 2.6. Three-Dimensional Reconstruction of Astrocytes

For three-dimensional reconstruction of astrocytes, we used dedicated software (Neurolucida, Microbrightfield, Williston, VT, USA) to store the coordinates of points of interest. To avoid ambiguity in the identification of objects of interest and provide greater accuracy in the reconstruction, the 4.0x objective was replaced by a high-power objective, the Plan Fluor 100x (NA 1.3; DF = 0.2 *μ*M, Nikon, Japan). A total of 171 astrocytes were reconstructed, 12 to 14 for each animal, all from the DG polymorphic layer.

We adopted a random and systematic sample procedure in the same sections analyzed by the optical fractionator, as has been suggested [44]. Astrocytes were selected from boxes of 70 × 70 *μ*m, separated from each other by 200 *μ*m intervals. Only astrocytes located within the box boundaries were selected for analysis. In rare exceptions, when no cells inside the box met the standard immunostaining and integrity requirements of the branches, astrocytes were chosen that were located near the edge of the box. We applied correction for shrinkage related to histological processing for all experimental groups but only for the *Z*-axis, with an assumption of 75% shrinkage, as previously suggested [45].

To assess morphological changes in astrocytes associated with murine prion disease, we measured and compared 14 morphological parameters of astrocytes reconstructed from the DG polymorphic layer of NBH and ME7-infected animals. Thus, each reconstructed astrocyte was the subject of multiple measures. Detailed descriptions of these 14 parameters (segment length, *μ*m; total number of endings; segment surface area, *μ*m^2^; segment volume, *μ*m^3^; segments/mm; fractal dimension, *k*-dim; branch node total; segment number; complexity; tree number; soma area, *μ*m^2^; soma perimeter, *μ*m; convex hull, *μ*m^3^) can be found elsewhere [46].

### 2.7. Statistical Analysis

The results from the Stereo Investigator, Neurolucida, and Any-Maze program were statistically analyzed using BioEstat 5.3 and GraphPad Prism software [47], applying parametric tests and multivariate analyses to establish differences or similarities among groups. A *p* < 0.05 was considered to indicate significance. The results are presented as arithmetic means and standard error values.

## 3. Results

### 3.1. Behavioral Changes

**Figure 2:**
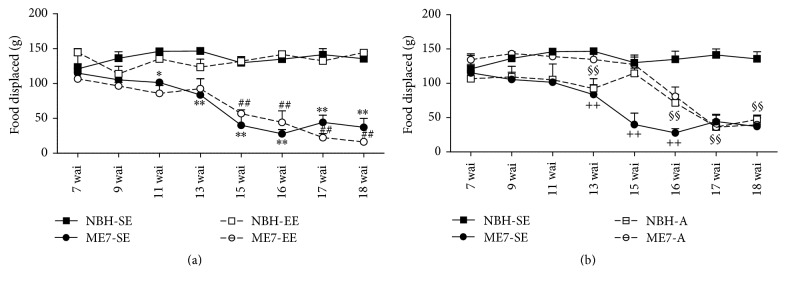
Burrowing activity. (a) Amount of burrowed food in the ME7-SE group compared to NBH-SE controls. Note significant reduction at 11 wai in ME7-SE compared with NBH-SE and at 15 wai in ME7-EE compared with NBH-EE. (b) ME7-SE young mice were different from ME7-A mice between 13 and 16 wai, and NBH-A showed a reduction in burrowing activity at 16 wai. (^*∗*^*p* < 0.05, ^*∗∗*^*p* < 0.01 versus NBH-SE; ^##^*p* < 0.01 versus NBH-EE; ^++^*p* < 0.01 versus ME7-A; ^§§^*p* < 0.01 versus NBH-SE; Two-way repeated measures Bonferroni posttests).

Our findings related to the environmental influence on prion disease progression are limited to young mice whereas those related to age influence are limited to mice raised on standard cages. ME7-SE but not ME7-A mice showed typical behavioral changes with disease progression [37]; however, as compared to young mice from standard cages, ME7-EE young animals seemed to exhibit slower disease progression.  Figure 2 shows the amount of burrowed food as a function of time progression. Two-way repeated measures ANOVA revealed significant interaction between infection and temporal progression (Figure 2(a), *F* = 6.39, *p* < 0.0001; and Figure 2(b), *F* = 5.71, *p* < 0.0001). EE influenced burrowing activity of prion-diseased mice; indeed, compared to NBH control groups, the amount of burrowed food in ME7-SE infected animals dropped significantly at 11 wai, but ME7-EE mice showed significant reduction only at 15 wai (Figure 2(a)). However, one-way repeated measures ANOVA revealed significant differences in burrowing between 7 and 15 wai for both ME7-SE and ME7-EE animals. No change in burrowed food was seen in control groups (NBH-SE and NBH-EE).

In contrast with young adult ME7-SE mice which show significant differences in burrowing activity, two-way repeated measurements ANOVA showed that aged mice ME7-A group did not differ in burrowing activity from age-matched controls (NBH-A). However, significant differences between ME7-A and ME7-SE at 13, 15, and 16 wai and between NBH-A and NBH-SE at 13, 16, 17, and 18 wai were found as an effect of aging (Figure 2(b)). One-way repeated measures ANOVA revealed significant differences between 7 and 16 up to 18 wai in ME7-A and between 7 and 17 up to 18 wai in NBH-A. For detailed statistical data, see Table S1 in Supplementary Material available online at https://doi.org/10.1155/2017/4504925.

Taken together, our findings suggest that prion-diseased young adult mice progressively reduced burrowing activity starting at 11 wai and that EE postponed this decrease by about 4 weeks, whereas aged mice, independently of infection, reduced significantly burrowing activity without significant differences in the amount of burrowed food between NBH-A and ME7-A individuals.

#### 3.1.1. Spatial Memory and Object Identity Recognition

**Figure 3:**
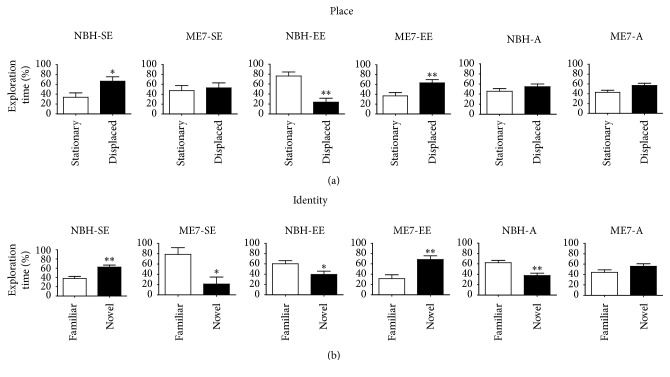
Object recognition impairments. At 17 wai, all mice were tested on object recognition tasks to recognize placement and identity of objects. (a) ME7-SE, NBH-A, and ME7-A did not distinguish displaced from stationary objects. All other groups succeeded in this task. (b) In the object identity recognition task, except for ME7-A, all groups recognized the identity of the objects (^*∗*^*p* < 0.05, ^*∗∗*^*p* < 0.01; two-tailed *t*-tests).

Figure 3 shows that significant differences were identified between time spent on exploration of displaced and stationary objects in the object placement test (Figure 3(a)). Significant differences between the amount of time spent on the exploration of familiar and novel objects were used as an indicator of object recognition in the object identity test (Figure 3(b)). Note that, on average, ME7-SE, NBH-A, and ME7-A could not distinguish displaced from stationary objects. All other groups succeeded in this task. However, all groups except ME7-A recognized the identity of the objects (Figure 3(b)), suggesting that the disease and aging first impair spatial memory task performance. At least in this time window, prion-diseased mice had their object identity recognition preserved. Although control aged mice could not distinguish between stationary and displaced objects, they distinguished between familiar and novel objects, whereas prion-diseased aged mice lost those abilities. For statistical details, see supplementary Table S2.

### 3.2. Prion Disease Pathology and Astrocytes Morphology at 18 wai

**Figure 4:**
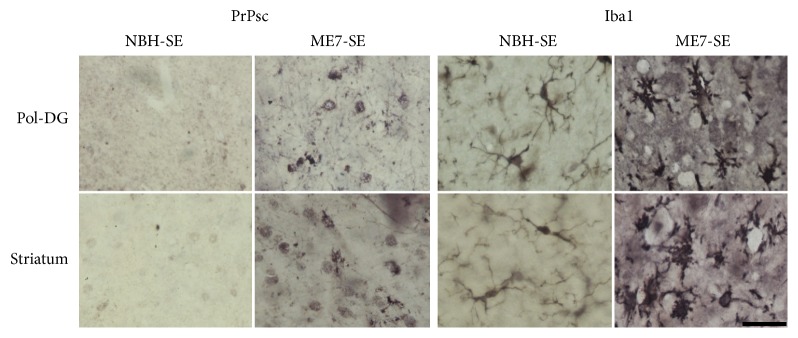
Photomicrographs from PrPSc and Iba1 immunolabeled sections from the polymorphic layer of dentate gyrus and striatum to illustrate mouse prion disease associated histological changes. Note PrPSc deposits and morphological activated microglia and vacuolation in ME7-SE. Scale bar: 25 *μ*m.

**Figure 5:**
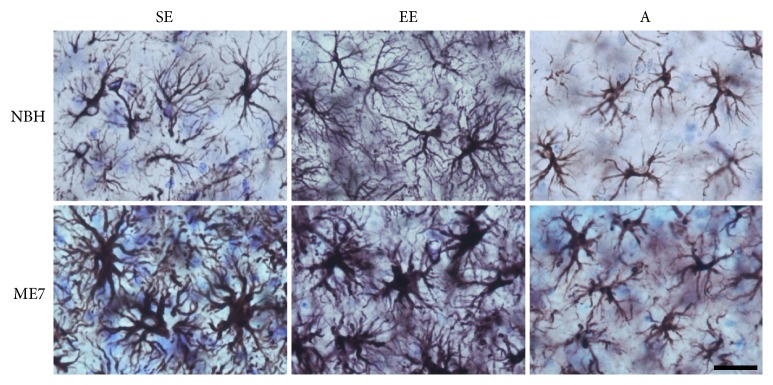
Photomicrographs from control and prion-diseased mice at 18 wai to illustrate morphological astrocytic changes in the stratum radiatum of CA3 from young and aged adults from standard and enriched environments. Scale bar: 25 *μ*m.

Stereological estimation and three-dimensional morphometric analysis of astrocytes were used to detect reactive astrocytosis in prion-diseased mice. At 18 wai histological signs of prion disease appeared in areas of injection, stereological, and morphometrical analyses, including PrPSc deposition, inflammatory microglial response, vacuolation (Figure 4), and reactive astrogliosis (Figure 5).

Reactive astrocytosis is clearly evident in prion-diseased young mice (ME7-SE and ME7-EE) but not in prion-injected aged mice (ME7-A). As compared to young mice, astrocytes from aged groups (NBH-A and ME7-A) are shrunk (Figure 5).

**Figure 6:**
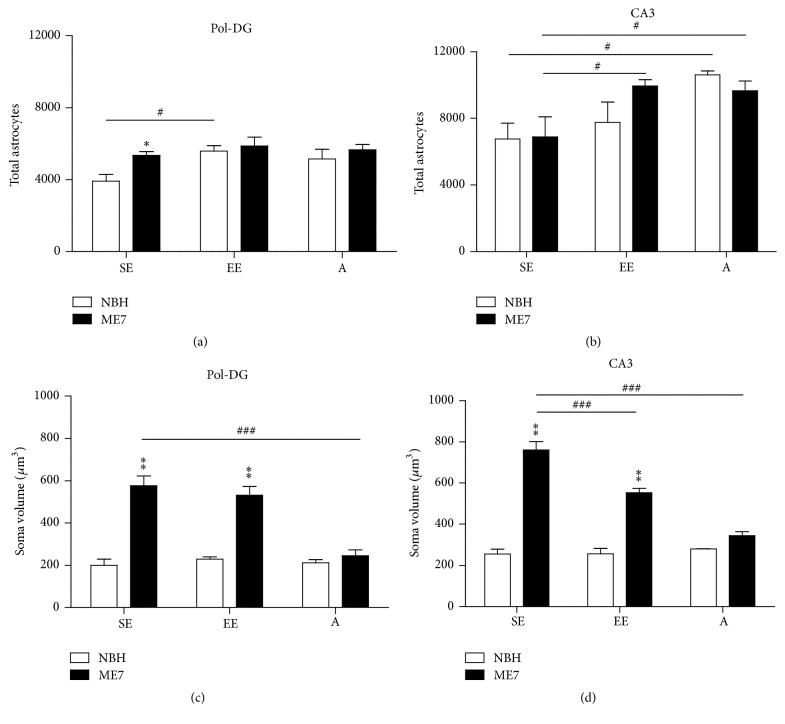
Astrocyte number and cell body volume changes. (a) In the polymorphic layer, ME7-SE mice showed higher number of astrocytes than NBH-SE animals. AS compared with NBH-SE, an increased number of astrocytes is observed in NBH-EE. (b) In the stratum radiatum of CA3, NBH-A and ME7-A mice showed higher number of astrocytes than NBH-SE and ME7-SE animals, respectively. In contrast to the polymorphic layer, stratum radiatum of CA3 of ME7-EE showed a higher number of astrocytes than that of ME7-SE mice. (c) and (d) show prion disease influence on the cell body volumes of astrocytes in the DG polymorphic layer and stratum radiatum of the CA3, respectively. ME7-SE and ME7-EE showed significant soma hypertrophy in astrocytes from DG and CA3 compared with their respective controls. However, ME7-A cell body volume was smaller than ME7-SE in the polymorphic layer and stratum radiatum of CA3 layers. In the stratum radiatum of CA3, the cell body volume of astrocytes from ME7-EE mice was smaller than that of ME7-SE animals (^*∗*, #^*p* < 0.05; ^*∗∗*^*p* < 0.01; ^###^*p* < 0.001 Bonferroni posttests).

We compared control and ME7-infected animals from different environments or different ages to detect potential alterations in astrocytes (Figures 6 and 8).

Figure 6 shows in young adult mice the environment (*F* = 10.02; *p* = 0.011) and disease (*F* = 6.02; *p* = 0.036) and influences on the total number of astrocytes in the DG polymorphic layer and stratum radiatum of CA3 (environment, *F* = 5.23; *p* = 0.045), with specific comparisons between groups given in Supplementary Table S1.

In the polymorphic layer, ME7-SE mice had higher number of astrocytes than NBH-SE animals (5342 ± 227.5 versus 3912 ± 376.3) whereas nonsignificant differences were detected in the comparisons between ME7-EE and NBH-EE (Figure 6(a)). However, as compared with SE, EE increased the number of astrocytes in the polymorphic layer in controls (NBH-EE versus NBH-SE). In the stratum radiatum of CA3, aged mice showed a significantly higher number of astrocytes than young adults (Figure 6(b)).

Different from the polymorphic layer, the stratum radiatum of CA3 of ME7-EE showed a higher number of astrocytes than that of ME7-SE mice whereas nonsignificant differences were detected in the comparisons between ME7-SE and NBH-SE or ME7-A and NBH-A animals (Figure 6(a)). The disease condition increased astrocyte cell body volume in ME7-SE and ME7-EE groups in the DG polymorphic layer and stratum radiatum of the CA3 (Figures 6(c) and 6(d)). However, EE reduced this hypertrophic effect on cell body astrocytes of ME7-EE group in the stratum radiatum of CA3. Prion disease did not produce volume changes in cell bodies in aged mice (ME7-A versus NBH-A); coherently, as compared with young infected mice cell body volumes from aged infected mice are smaller (ME7-A versus ME7-SE) (Figure 6(d)). A significant interaction between disease and environment (*F* = 11.77; *p* = 0.006) and between disease and age (*F* = 62.02; *p* < 0.0001) was found in CA3 astrocytes cell body volumes. See Supplementary Table S3 for more details.

**Figure 7:**
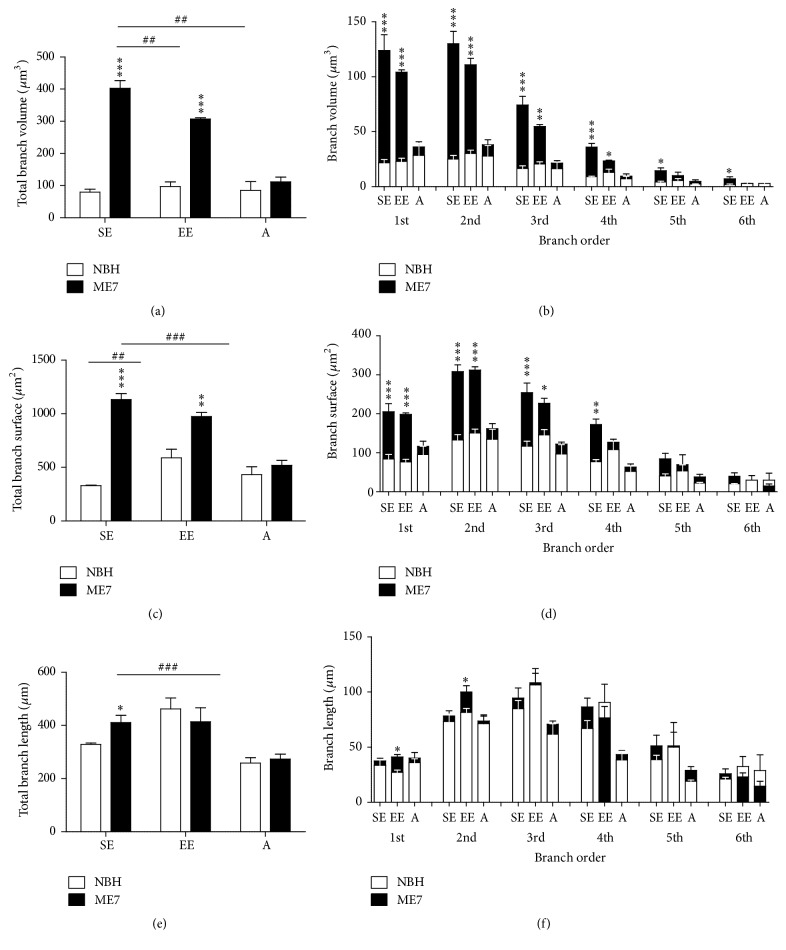
Astrocyte morphometry. (a) ME7-SE and ME7-EE mice showed significantly higher branch volumes compared to NBH-SE and NBH-EE animals, respectively. ME7-EE and ME7-A groups had reduced branch volumes versus ME7-SE mice. (c) Prion disease also increased the surface area of astrocyte branches compared with NBH-SE and ME7-A groups. An increase in branch surface area also was seen in ME7-EE versus NBH-EE mice and in NBH-EE versus NBH-SE animals. (e) ME7-SE astrocytes showed significantly longer branches than NBH-SE and ME7-A astrocytes. (b, d) Total volume and total surface area in ME7-SE mice were found in the 1st, 2nd, 3rd, 4th, 5th, and 6th branch orders whereas, in EE animals, effects on surface area were limited to branches from the 1st to 3rd orders, and volume differences were limited to between the 1st and 4th orders. (f) 1st and 2nd branch orders from ME7-EE were significantly longer than the corresponding branches of ME7-SE. Nonsignificant alterations were detected in aged mice (^*∗*^*p* < 0.05; ^*∗∗*, ##^*p* < 0.01; ^*∗∗∗*, ###^*p* < 0.001 Bonferroni posttests).

Figure 7 shows graphic representations of astrocyte morphological changes on astrocyte three-dimensional reconstructions of the polymorphic layer at 18 wai. The total volume of branches was influenced by prion disease (*F*_(1,10)_ = 245.1; *p* < 0.0001), EE (*F*_(1,10)_ = 5.19; *p* = 0.046), and aging (*F*_(1,11)_ = 49.36; *p* < 0.0001) with significant interactions between these variables (prion and environment: *F*_(1,10)_ = 10.91; *p* = 0.008; prion and aging: *F*_(1,11)_ = 53.10; *p* < 0.0001). In contrast, the surface area of branches was affected only by prion disease independent of environmental condition (*F*_(1,10)_ = 96.74; *p* < 0.0001) and by aging (*F*_(1,11)_ = 22.72; *p* = 0.0006), with a significant interaction between prion disease and aging (*F*_(1,11)_ = 44.76; *p* < 0.0001). The total length of branches was influenced by prion disease (*F*_(1,11)_ = 5.039; *p* = 0.046) and aging (*F*_(1,10)_ = 23.21; *p* = 0.0005). In agreement, two-way ANOVA Bonferroni posttests (Table S3) revealed, as compared with NBH-SE and NBH-EE, significantly higher branch volumes in the ME7-SE and ME7-EE animals. ME7-EE and ME7-A groups showed reduced branch volumes compared to ME7-SE animals (Figure 7(a)). As compared with NBH-SE and ME7-A groups, ME7-SE showed an increase in the surface of the branches. Similarly, as compared with NBH-EE, an increase in the branch surface area was found in ME7-EE mice. A similar increase in branch surface areas was also found in NBH-EE in comparison with NBH-SE (Figure 7(c)). On average, the branches of the ME7-SE group were significantly longer than those from the NBH-SE and ME7-A groups.

Differences in branch volumes between NBH and ME7 started at the first-order branches and persisted until the sixth order in SE groups but only until the fourth order in EE animals (Figure 7(b)). Prion disease also seemed to affect the surface of more distal branches of the SE group (fourth order) compared to the EE group (third order) (Figure 7(d)). Although no differences were observed in the total length of these branches, the first- and second-order branches were longer in ME7-EE in comparison with NBH-EE mice (Figure 7(f)). For more details, see supplementary Table S3.

**Figure 8:**
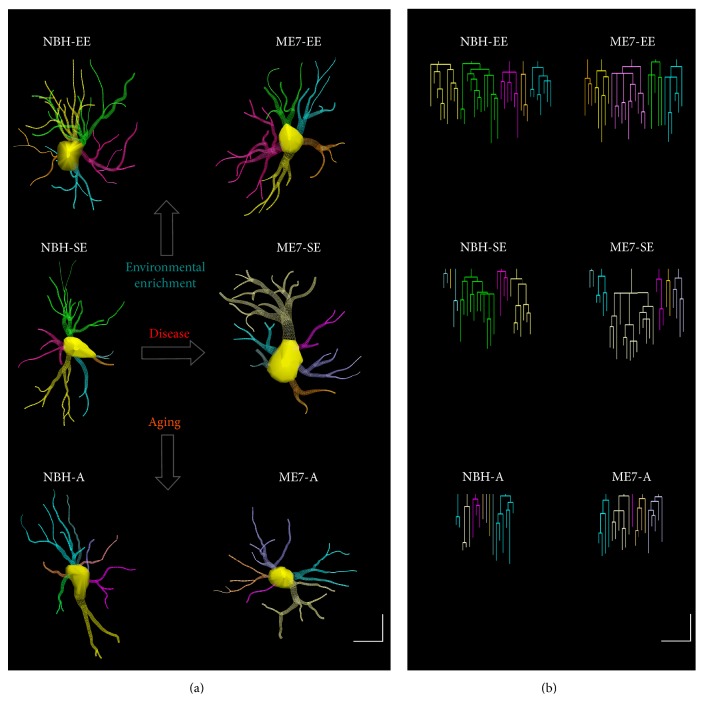
Three-dimensional reconstructions of astrocytes of the DG polymorphic layer at 18 wai (a) with corresponding dendrograms (b) in prion-diseased mice and age-matched controls. Note that aging seemed to shrink astrocyte arbors and that prion disease seemed to be associated with thicker astrocyte processes and larger cell bodies. EE was associated with an increase in the number of branches and a reduction in the prion disease-induced hypertrophy. Linear dendrogram of each astrocyte arbor with the length of each branch segment is displayed to scale as vertical lines and sister branches is horizontally displaced. Arrows indicate changes induced by each variable (environment, infection, and age). Dendrogram was plotted and analyzed with Neuroexplorer (MicroBrightField). Branches of the same parental (primary branch) trunk are shown in one color. Scale bar: 10 *μ*m.

Figure 8 shows graphical models of three-dimensional reconstructions of astrocytes of the polymorphic layer of the DG with correspondent dendrograms, to compare astrocytes from prion-diseased mice at 18 wai with those of control mice to measure possible influences of environment and age on astrocyte morphology. To illustrate morphological changes, the mean values of multiple measurements of branches and soma astrocytes were previously estimated, and the selected reconstructions exhibited metric features close to the mean values of the mean astrocyte of each experimental group. Note that, as compared to control mice, prion-diseased animals show a remarkable increase in the soma and branch volumes of astrocytes.

**Figure 9:**
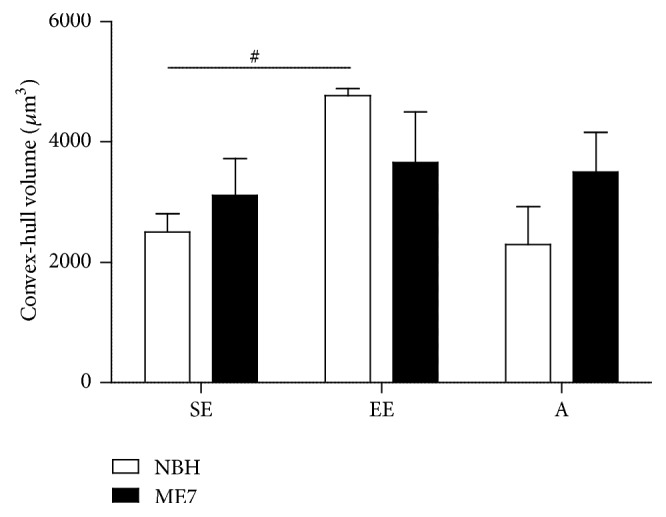
Graphic representations of convex-hull analysis showing that EE increased by as much as 91% the total tree field volume of the astrocytes from NBH-EE as compared with NBH-SE mice (^#^*p* < 0.05; Bonferroni posttest).

Figure 9 shows graphic representations of convex-hull analysis applied to the reconstructed astrocytes from the DG polymorphic layer at 18 wai. As compared with NBH-SE mice, EE increased by as much as 91% the total tree field volume of the astrocytes (NBH-EE versus NBH-EE = 4778.95 ± 110.85 versus 2500.77 ± 306.17; *t* = 2.74, *p* < 0.05; two-way ANOVA, *F*_(1,9)_ = 6.14; *p* = 0.035).

**Figure 10:**
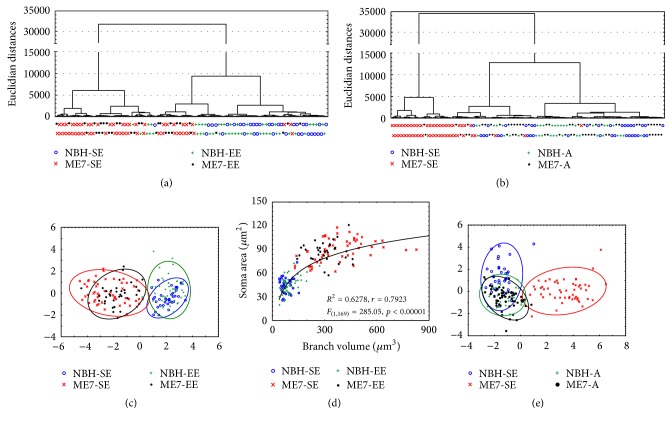
Graphic representation of results of multivariate statistical analysis of morphometric features of all reconstructed astrocytes (*n* = 269). (a) Dendrogram illustrating astrocyte morphological phenotypes of the DG polymorphic layer from infected and control adults. Branch length, nodes, and soma area were the morphological features that most contributed to cluster formation in young adult groups. (b) Dendrogram illustrating astrocyte morphological phenotypes of the DG polymorphic layer from infected and control aged mice. Tree surface, soma area, branch length, branch nodes, and tree volumes were the morphological features that most contributed to the cluster formation of SE young adults and aged groups. (c) Canonical distribution of the discriminant analysis of morphological phenotypes of the polymorphic layer from both control and infected adult mice. Canonical analysis based on these morphological features revealed significant Mahalanobis distance between ME7 and NBH astrocytes and (e) between ME7-SE and all others. (d) Significant logarithmic correlation was detected between branch volumes and soma area, suggesting an interdependence between these morphological features.

Figure 10 shows graphical representations of cluster analysis of all reconstructed astrocytes (*n* = 269) to test the hypothesis that astrocytes from prion-diseased mice (ME7-SE, ME7-EE, and ME7-A) are morphologically distinct from control mice astrocytes (NBH-SE, NBH-EE, and NBH-A). Branch length, number of branch nodes, and soma area were the morphological features that most contributed to the cluster formation of young adult groups (Figure 10(a)). A canonical graphic representation of the discriminant analysis based on these morphological features revealed significant interception between ME7 astrocytes and between NBH-SE and NBH-EE mice. Except for one individual from NBH-EE, included in the zone of interception of ME7-SE and ME7-EE, no other interceptions were observed between NBH and ME7 individuals (Figure 10(c)). Significant logarithmic correlation (*R*^2^ = 0.63, *F* = 285.05, and *p* < 0.00001) was detected between branch volumes and soma area, suggesting an interdependence between these morphological features (Figure 10(d)). Tree surface, soma area, branch length, branch nodes, and tree volume were the morphological features that most contributed to the cluster formation of aged groups (Figure 10(b)). A canonical graphic representation of the discriminant analysis revealed significant interception between ME7-A, NBH-A, and NBH-SE astrocytes, with significant differences in Mahalanobis centroid distance (*F*_(7,177)_ = 69.89, *p* < 0.0001) from the ME7-SE astrocytes (Figure 10(e)).

**Figure 11:**
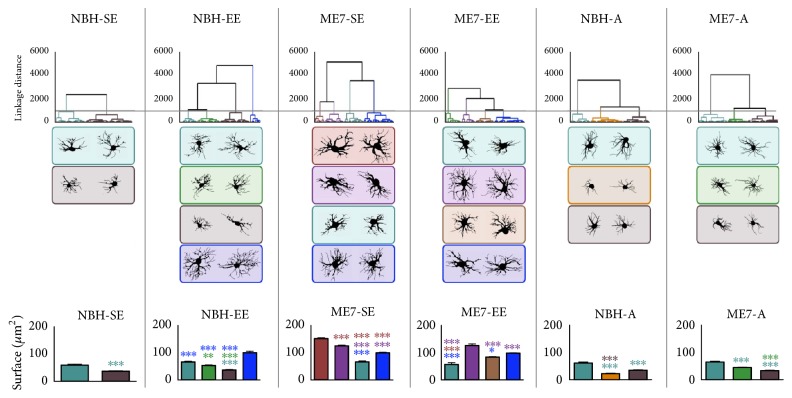
Hierarchical cluster analysis of morphological features of astrocytes from the polymorphic layer limited to each experimental group. Astrocytes associated with SE exhibited only two morphological families, whereas those from EE animals showed four distinct morphological families. Astrocytes from ME7 groups were distributed into four different families with a larger surface area, and two of these astrocyte families exhibited significantly higher values for branch surface areas than all NBH families. Aged groups showed three astrocyte families. NBH-A formed a family with smaller surface area, suggesting that SE and aging, acting together, shrank astrocytes trees. In contrast, prion disease increased it. Branch surfaces (from all groups), soma area (from ME7 groups), and branch length (from EE and NBH-A groups) were the morphometric features that most contributed to cluster formation. (^*∗*^*p* < 0.05; ^*∗∗*^*p* < 0.01; ^*∗∗∗*^*p* < 0.001, Bonferroni posttests).

Figure 11 shows graphical representations of cluster analysis of each experimental group (NBH-SE, NBH-EE, ME7-SE, ME7-EE, NBH-A, and ME7-A) to test the hypothesis of occurrence of astrocyte morphological families inside each group. Discriminant analysis and two-tailed *t*-tests of morphometric features indicate the morphometric features that most contribute to cluster formation and distinguish astrocytes from different morphological families. Note that astrocytes from ME7 groups tended to have larger somas and thicker branches than astrocytes from control groups. Control groups from the SE animals exhibited only two morphological families of astrocytes whereas EE mice showed four distinct morphological families. A third family was found in NBH-EE, showing larger astrocyte trees (surface area) than the other three families. ME7 groups showed in general larger soma and branch surfaces than control mice, distributed into four different families where astrocyte families exhibited larger surface area than control mice astrocyte families. In addition, two of these astrocyte families from ME7 mice exhibited significantly higher values for branch surface areas than all NBH families. Different from ME7-SE, the ME7-EE group showed only one family with higher surface area than the control NBH-EE group. Aged groups also had three morphometrically distinct families of astrocytes, and the NBH-A group had the family with the lower surface area. Branch surfaces (all groups), soma area (ME7 groups), and branch length (EE and NBH-A groups) were the morphometric features that most contributed to cluster formation.

**Figure 12:**
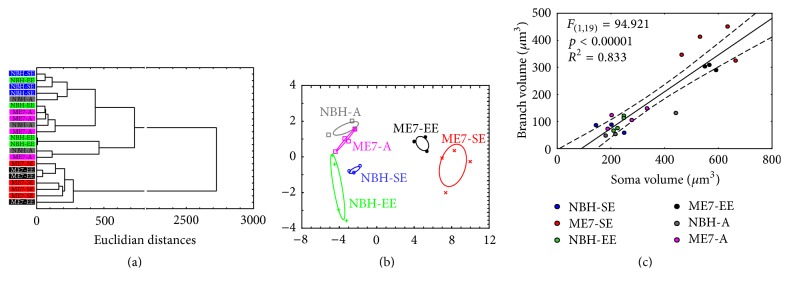
Astrocyte morphological changes in prion-diseased adult and aged mice. (a) Cluster analysis and (b) canonical distribution of discriminant analysis. Note the clear distinction between NBH and ME7 young adults in both cluster and discriminant canonical analysis. Branch volume was the morphometric feature that most contributed to the cluster formation. However, ME7-A and NBH-EE control mice occupy the same cluster in the dendrogram, suggesting that prion disease in aged mice did not alter astrocyte morphology as it did in adult mice. (c) Soma volumes estimated by the nucleator method are linearly correlated with branch and volumes.

Astrocyte morphological changes in prion-diseased young adult and aged mice are illustrated in Figure 12. Cluster analysis and canonical distribution of discriminant analysis revealed a clear distinction between NBH and ME7 young adults. Branch volume was the morphometric feature that most contributed to the cluster formation. However, ME7-A and NBH-EE control mice could not be distinguished from one another, suggesting that prion disease in aged mice did not change astrocyte morphology in the same proportion as it did in younger mice. Soma volumes estimated by the nucleator method were linearly correlated with branch surfaces and volumes (*R*^2^ = 0.83; *F*_(1,19)_ = 94.92; *p* < 0.00001), suggesting interdependence between these variables.

## 4. Discussion

The neuropathology of prion disease has been widely investigated in experimental models, contributing new understanding about cellular and molecular mechanisms of chronic neurodegeneration. However, most of previous contributions to understand neuropathology and clinical signs and subjacent neuropathology of prion disease were done at late stages of the disease [5] when motor impairments and posture changes are evident [48, 49]. Before clinical signs (used to define the incubation period), preclinical signs may be apparent including reduction in burrowing activity, anhedonia, and significant increase of open field activity [50]. Burrowing is by far the most sensitive task to detect early hippocampal dysfunction in mouse prion disease which coincides with preclinical stage onset [34]. In this study, we combined three-dimensional reconstruction and an unbiased stereology sampling approach to quantify morphological changes of astrocytes in young and aged diseased animals in the preclinical stage (18 wai) to investigate the hypothesis that EE would slow prion disease progression. Our findings from the DG and CA3 revealed that astrocytes from prion-diseased animals show numerical and morphological differences when compared to control animals. The DG polymorphic layer is targeted by reactive astrocytosis in ME7 groups, showing an increase in cell body volume and number of astrocytes, while in CA3, prion-induced changes were limited to cell body hypertrophy defined as significant increase in the volume of soma. The three-dimensional morphometric analysis of astrocytes from the polymorphic layer of infected animals also revealed significant hypertrophy of proximal and distal segments and significant changes in the branching pattern of astrocytes. EE control animals showed significant changes in morphology of astrocytes from the polymorphic layer by increasing the number of branches and the volume of parenchyma covered by astrocyte trees. In addition, EE was associated with increased number and GFAP immunostaining of astrocytes. As compared with prion-diseased SE animals, infected EE mice showed a reduction in the increase in CA3 astrocytic cell bodies, mimicking the effects of EE on astrocyte process volume and spatial distribution in the polymorphic layer of infected animals. In the CA3, aged mice also showed a higher number of astrocytes and significant cell body atrophy in the DG polymorphic layer and CA3. The branches of astrocytes were not significantly altered in aged mice, and prion disease did not affect their morphology. Multivariate statistical methods revealed morphologically distinct subtypes of astrocytes in each experimental group, but the molecular mechanisms and functional implications of such changes remain to be investigated. In the present report we found that ME7-SE, NBH-A, and ME7-A failed to recognize object placement and only ME7-A failed to recognize object identity at 18 wai. The one-trial object recognition task involves memory of a familiar object in parallel with the detection and encoding of a novel object. It has been largely suggested that novelty preference concept should be used to interpret identity and placement object recognition in many relevant studies, including mice and rats [37, 39, 40, 51]. Indeed, a normal mouse when exposed to a familiar object alongside a novel object frequently spends more time exploring the novel than the familiar object and this has been indicated that a memory of the familiar object is available [38]. However, our findings indicate that NBH-EE spent more time in the stationary object than in displaced object and in the object recognition identity test ME7-SE, NBH-EE, and NBH-A spent more time in the familiar object than the new object.

Because these results are unexpected outcomes for this type of tests previous reports suggest they could be associated with neophobia and anxiety-like behavior [52, 53]. However, the validity of this concept has been recently questioned and the novelty preference model and novelty-bias hypothesis proposed to account for object recognition memory have been reexamined [38, 54]. In addition, neophobia as an explanation of rodents preference for familiar objects has been questioned [54]. To minimize neophobic behavior, we applied in the present report the object recognition protocol described elsewhere [39] in which animals are handling for 7 consecutive days and then introduced to the test apparatus for several sessions of habituation (5 days) without and with objects before the start of the object recognition test. If we succeeded in our attempts to reduce neophobia, it remains a temptation to speculate that significant differences between times spent in the objects (either novel or familiar) are not a consequence of neophobia and do require object recognition (to recognize familiar object) or the absence of familiarity (to identify new object). In both cases the ability to recognize familiar objects or detect novelty should be intact. Another important issue is related to the fact that aged infected mice did not distinguish object identity. Is this impairment related to prion disease or is this a consequence of aging? Because control aged mice distinguish object identity, it may be possible that prion disease could be associated with this deficit. However, because both normal and prion-diseased aged mice reduced at the same time window burrowing activity it is reasonable to suggest they may have both hippocampal dysfunction [34]. Whether or not these hippocampal dysfunctions have the same cause remains to be investigated.

### 4.1. Astrocytic Changes in Albino Swiss Mouse Model of Prion Disease

Astrocytes exhibit typical morphological changes when they are reactive, and the main feature of these changes is an increase in GFAP expression; to our knowledge, this study is the first to describe morphological changes in astrocytic three-dimensional reconstruction in an experimental mouse model of prion disease using a stereological sampling approach to select astrocytes for reconstruction. A single quantification of the total number of astrocytes in experimental models of prion disease by stereological methods was performed previously [27], and the majority of available data are limited to two-dimensional quantification and qualitative analysis. Our previous results [27] and present findings revealed a significant increase in GFAP-immunolabeled astrocytes in the DG polymorphic layer at 18 wai with ME7-infected brain homogenate in both hippocampal formation and dorsal striatum. Although previous findings indicated increased GFAP synthesis as the main protein affected over the time course of prion disease [4], no significant numerical increase in astrocytes was detected by studying cells with double staining for Ki67 and GFAP at 21 wai with ME7-infected brain homogenate in the hippocampus. It remains to be investigated if these differences in GFAP results are related to the adoption of distinct experimental models or technical procedures.

In response to CNS inflammation astrocytes may adopt neurotoxic or neuroprotective functional phenotypes [55]. In the present report the cluster analysis based on surface area (discriminant variable) suggested that astrocytes from polymorphic layer of dentate gyrus of young adult ME7 infected groups include 4 morphological distinct families. The highest values of surface area indicating thicker astrocytes branches correspond to an increase in GFAP expression. Because pro- and anti-inflammatory gene expression induces an increase in glial fibrillary acidic protein expression we do not know, limited to morphological phenotypic analysis, in what direction (neurotoxic or neuroprotective) astrocytic functional polarization is oriented for [56]. Similarly control animals from enriched environment showed two more astrocytic families than controls from standard environment. Although it is reasonable to suggest that environmental enrichment may induce neuroprotective phenotypes, morphological analysis alone is not enough to confirm this hypothesis.

### 4.2. Relations among Prion Disease and Reactive Astrocytosis in the DG, EE, and Cognitive Function

In homeostatic conditions, the memory formation of recent events is entirely dependent on DG. Indeed, the dendrites of the granular neurons of the DG are the main target of the entorhinal-to-DG projection, and placement of these synapses in the DG molecular layer is the first step in episodic memory formation [57]. The integrity of the DG but not the CA1 is essential to the discrimination of similar objects in different contexts [58]. Given this functional architecture, it is reasonable to propose that astrocyte changes associated with the DG in sick animals may contribute directly to breaking the homeostatic balance of the neuronal microenvironment, alter synaptic function, and compromise the neurogenesis of the subgranular layer. In fact, from the data gathered in this study, it becomes apparent that behavioral changes worsen over the time course of the disease and that this worsening is evident from the performance on hippocampal-dependent tasks. The first signs of hippocampal-dependent sickness that are behavior dependent are observed in burrowing activity tests, nest building, and consumption of glucose, all arising around 12 wai, while motor changes such as open field exploration and muscle strength show signs of change around 18 wai [35]. Our results show that the ME7 injection in the dorsal striatum did not change the temporal course of the disease compared to prion disease induced by hippocampus injection. Injection in both places showed clear hippocampal degeneration and corresponding changes in burrowing activity at 11 wai for SE mice and at 15 wai for EE mice. Previous studies have shown that, regardless of the route of inoculation of the prion agent, the hippocampus is one of the main targets of chronic neurodegeneration associated with the disease [59, 60], and the burrowing test is sensitive and specific to hippocampal damage [34, 61]. As far as we know, our results are the first to demonstrate a spatial memory decline in object recognition associated with reactive astrogliosis in a murine model of prion disease. We are also the first to demonstrate a reduction in the rate of prion disease progression in EE mice.

Physical exercise and multisensory stimulation have been applied as effective nonpharmacological interventions to decrease the rapidity of AD progression. EE is widely used in experimental models in many neurodegenerative diseases, resulting in a growing number of publications investigating the cellular and molecular mechanisms underlying the neuroprotective effects it may confer [14, 15]. Because previous studies have not been conducted to investigate the impact of EE in prion disease, we are left with the comparison with other experimental models using transgenic mice models for AD maintained in EE [16, 17]. In AD (PDAPP-J20) model, EE decreases the volume of GFAP-immunolabeled astrocytes [38]. Similarly, these findings were later confirmed in another transgenic mouse model of AD (3xTg-AD) which showed that mice from EE had a significant decrease in the surface and volume of cell bodies and branches of astrocytes [62]. Our findings are in line with both observations.

In addition, our findings expand previous results and contribute to the understanding of astroglial morphological plasticity in healthy animals exposed to an EE. Indeed; we investigated previously whether aging and environmental-related influences on learning and memory were correlated with astroglial changes in the DG [23]. We demonstrated that episodic-like memory was absent in mice raised in impoverished conditions but was preserved in both young and aged EE mice and these results were associated with a laminar-dependent increased number of astrocytes in both aging and EE. Thus, impoverished conditions seem to be associated with abnormal cognitive development and an altered laminar distribution of astrocytes in the DG [23].

Therefore, we earlier suggested that astrocytosis in different conditions may arise from different astrocyte phenotypes. Here, we expanded our previous results, which were limited to the molecular layer of the DG, to the polymorphic layer, where we also found an increased number of astrocytes, increased number of branches, and increased volume of the neuropil covered individually by each astrocyte. These results can be compared to those of Viola et al. (2009), in which the two-dimensional reconstruction of astrocytes from the stratum radiatum of CA1 showed increased branching after 8 weeks of EE [25].

Similarly, Sampedro-Piquero et al. [26] demonstrated that improvement in cognitive performance in older EE mice may be related to an increase in GFAP selective immunoreactivity for astrocytes in the DG and CA3 and by increasing the length, number of intersections, and branching nodes of astrocytes of the DG, CA1, and CA3. Moreover, Beauquis and collaborators [62] used confocal microscopy and immunostaining for GFAP to show that CA1 astrocytes increased the branching pattern after 3 months of EE. In pathological conditions, astrocytes from EE animals started to produce neurotrophic factors such as BDNF and GDNF [63] and altered the immune response by promoting neuroprotective cytokine modulation.

Taken together, our results demonstrate for the first time in an albino Swiss mouse ME7 model of prion disease that EE produces reduction in the rate of cognitive decline measured with hippocampal-dependent tasks which was associated decreased reactive astrocytosis. However, in the present report, whereas most young mice revealed both preclinical and neuropathological signs of prion disease, aged mice failed to develop these features. Thus, in contrast to old age, young age may be a risk factor for infectious prion disease following exposure to prion agent [15]. Our findings confirm and expand other reports demonstrating that, in contrast to adult young mice, which succumbed to clinical prion disease, no aged mice exposed to prion agent developed clinical disease [13, 14]. Different though from previous descriptions demonstrating that both aged and young mice infected with scrapie agent directly into the brain were fully susceptible to disease and developed clinical scrapie with similar incubation periods [13], our findings did not reproduce these results. Indeed, in contrast with young adult mice, which showed the full spectrum of clinical and neuropathological signs of prion disease, at least until 18 wai, when all animals were euthanized, neither clinical nor neuropathological features of prion disease were found in ME7-infected aged mice. Because we used the same amount of the same infected ME7 brain homogenate, injected in the same stereotaxic coordinates, it is difficult to explain why albino Swiss aged mice did not become ill. Previous comparisons between prion disease models using albino Swiss mice and C57BL6 revealed that, on average, early behavioral changes in albino Swiss mice start 4 weeks later than in C57BL6 animals [27], so it may be possible that strain differences contributed to these contrasting results. Thus, our data suggest that when the ME7 agent is delivered directly to the brain of albino Swiss mice, host age can have a significant influence on the onset of clinical disease.

### 4.3. Technical Limitations

Based on morphological analysis of 3 selective astroglial markers (anti-GFAP, anti-glutamine synthetase, and anti-S-100ß) an emerging view that “astrocytes” constitute a heterogeneous population even within a given region became apparent [64]. Thus, the present report limited to GFAP-immunolabeled astrocytes may show only part of the potential morphological and numerical changes in mouse prion disease model, under influence of age and environmental changes. With such clear limitation in mind, it is worth recalling that previous studies, using stereology, have shown that age [23, 65] and environment [23] appear to induce changes in GFAP-immunolabeled astrocytes in the dentate gyrus. In addition, an enriched environment promoted changes in the astrocytes morphological phenotypes which appeared longer and more ramified [25, 26], stimulating neurogenesis and glycogenesis [66], increasing the network of GFAP-immunolabeled cells [63].

Previous reports using different animal lineages, different histological procedures, and counting methods revealed contradictory results related to the influences of age and environment on the number and morphology of astrocytes [23, 24, 26, 63]. To minimize most sources of nonbiological variation, we standardized all methodological procedures as previously suggested [42, 65]. In addition, due to mechanical factors associated with the vibratome sectioning and further dehydration procedure, a nonuniform shrinkage in the *z*-axis of the sections is obtained [67]. Thus, estimates of modifications in the *x*/*y* dimensions during tissue processing cannot be linearly extrapolated to the *z* dimension. These methodological constraints impose limitations that should be taken into consideration when analyzing the present data. However, it is important to note that an indication of a severe shrinkage in *z*-axis is the curling of branches, implying that individual processes did not shrink at the same rate as the slice in which they are located. This pattern was not observed in the reconstructed cells of our study. It was recently demonstrated that the final thickness in the *Z*-axis is approximately 25% of the cut thickness after dehydration and clearing [45] and therefore we applied 75% shrinkage correction along the *z*-axis and no corrections to the *x*/*y* dimensions. Finally, because microscopic 3D analysis is limited to a small fraction of the whole area of interest, sampling limitation is inevitable. However, as previously described [68], our morphometric analysis of astrocytes combined stereological sampling approach and three-dimensional reconstruction to guarantee that all regions of the areas of interest had the same probability to contribute to the sample of astrocytes three-dimensionally reconstructed.

## 5. Conclusion

Overall, our findings revealed the beneficial effects of EE in slowing prion disease progression and reducing neuropathological and behavioral outcomes. Moreover, they confirm previous results suggesting that host age is an important barrier to the full-spectrum manifestation of the clinical and neuropathological features of prion disease. Prion disease neuropathological features have been suggested to be similar to those of AD, but it is important to highlight that, in contrast to AD, a chronic neurodegenerative disorder of the elderly, prion disease does not appear to express its full severity in aged immune systems.

## Supplementary Material

Table S1. Environmental influences on burrowing activity in both prion diseased and control mice.
Table S2. Environmental influences on object recognition scores in both prion diseased and control mice.
Table S3. Environmental influences on number and morphometry of astrocytes in both prion diseased and control mice.

## Abbreviations

A: Aged
EE: Enriched environment
GFAP: Glial fibrillary acidic protein
Iba1: Ionized calcium-binding adapter molecule 1
ME7: Prion strain
NBH: Normal brain homogenate
SE: Standard environment
wai: Weeks after inoculation.
